# The structure of BVU2987 from *Bacteroides vulgatus* reveals a superfamily of bacterial periplasmic proteins with possible inhibitory function

**DOI:** 10.1107/S1744309109046788

**Published:** 2010-03-05

**Authors:** Debanu Das, Robert D. Finn, Dennis Carlton, Mitchell D. Miller, Polat Abdubek, Tamara Astakhova, Herbert L. Axelrod, Constantina Bakolitsa, Connie Chen, Hsiu-Ju Chiu, Michelle Chiu, Thomas Clayton, Marc C. Deller, Lian Duan, Kyle Ellrott, Dustin Ernst, Carol L. Farr, Julie Feuerhelm, Joanna C. Grant, Anna Grzechnik, Gye Won Han, Lukasz Jaroszewski, Kevin K. Jin, Heath E. Klock, Mark W. Knuth, Piotr Kozbial, S. Sri Krishna, Abhinav Kumar, David Marciano, Daniel McMullan, Andrew T. Morse, Edward Nigoghossian, Amanda Nopakun, Linda Okach, Christina Puckett, Ron Reyes, Christopher L. Rife, Natasha Sefcovic, Henry J. Tien, Christine B. Trame, Henry van den Bedem, Dana Weekes, Tiffany Wooten, Qingping Xu, Keith O. Hodgson, John Wooley, Marc-André Elsliger, Ashley M. Deacon, Adam Godzik, Scott A. Lesley, Ian A. Wilson

**Affiliations:** a Joint Center for Structural Genomics, http://www.jcsg.org, USA; b Stanford Synchrotron Radiation Lightsource, SLAC National Accelerator Laboratory, Menlo Park, CA, USA; c Wellcome Trust Sanger Institute, Wellcome Trust Genome Campus, Hinxton CB10 1SA, England; d Department of Molecular Biology, The Scripps Research Institute, La Jolla, CA, USA; e Protein Sciences Department, Genomics Institute of the Novartis Research Foundation, San Diego, CA, USA; f Center for Research in Biological Systems, University of California, San Diego, La Jolla, CA, USA; g Program on Bioinformatics and Systems Biology, Burnham Institute for Medical Research, La Jolla, CA, USA; h University of California, San Diego, La Jolla, CA, USA; i Photon Science, SLAC National Accelerator Laboratory, Menlo Park, CA, USA

**Keywords:** BVU2987, DUF2874, PF11396, human gut microbiome, β-lactamase inhibitor protein-like fold, putative inhibitor proteins

## Abstract

Proteins that contain the DUF2874 domain constitute a new Pfam family PF11396. Members of this family have predominantly been identified in microbes found in the human gut and oral cavity. The crystal structure of one member of this family, BVU2987 from *Bacteroides vulgatus*, has been determined, revealing a β-lactamase inhibitor protein-like structure with a tandem repeat of domains. Sequence analysis and structural comparisons reveal that BVU2987 and other DUF2874 proteins are related to β-lactamase inhibitor protein, PepSY and SmpA_OmlA proteins and hence are likely to function as inhibitory proteins.

## Abstract 2

The crystal structure of the BVU2987 gene product from *B. vulgatus* (UniProt A6L4L1) reveals that members of the new Pfam family PF11396 (domain of unknown function; DUF2874) are similar to β-lactamase inhibitor protein and YpmB.

## 1. Introduction

Recent interest in metagenomics (Sleator *et al.*, 2008 ▶), together with advances in genomic and proteomic techniques, has led to a rapid evolution in the study of the human gut microbiome (Frank & Pace, 2008 ▶; Ley *et al.*, 2008 ▶; Verberkmoes *et al.*, 2009 ▶) and its association with human health and disease (Mai & Draganov, 2009 ▶; Kinross *et al.*, 2008 ▶; Turnbaugh *et al.*, 2009 ▶; Ordovas & Mooser, 2006 ▶; Othman *et al.*, 2008 ▶; O’Keefe, 2008 ▶). The sequencing of complete genomes of bacteria from the human gut, such as *Bacteroides thetaiotaomicron* (Xu *et al.*, 2003 ▶) and *B. vulgatus* (Xu *et al.*, 2007 ▶), as well as from the oral cavity, such as *Porphyromonas gingivalis* (Nelson *et al.*, 2003 ▶), has identified many novel proteins of unknown function. Large-scale structure determination of these proteins can provide functional insights and may lead to the identification of new drug targets for therapeutic exploitation (Zaneveld *et al.*, 2008 ▶).

Towards this goal, the BVU2987 protein from *B. vulgatus* ATCC 8482, one of the predominant members of the human gut microbiome, was selected for crystallographic structure determination. BVU2987 is a 145-residue protein with a calculated pI of 5.36 and is annotated as a putative periplasmic protein based on the predicted N-­terminal signal peptide. The protein sequence has been assigned to a novel protein family that is predominately found in species that populate the human oral cavity and gut microbiomes, including *Bacteroides*, *Campylobacter* and *P. gingivalis* (the predominant agent of periodontal disease). Proteins in this family are annotated either as putative periplasmic proteins or as conserved hypothetical proteins, but none have been biochemically characterized. Analysis of our structure and of the available sequences shows that collectively this family forms part of a larger superfamily of bacterial periplasmic proteins that all adopt a fold similar to β-lactamase inhibitor protein (BLIP-like fold) and appear to share some broad spectrum of inhibitory function.

## 2. Materials and methods

### 2.1. Protein production and crystallization

Clones were generated using the Polymerase Incomplete Primer Extension (PIPE) cloning method (Klock *et al.*, 2008 ▶). The gene encoding BVU2987 (GenBank YP_001300247.1) was amplified by polymerase chain reaction (PCR) from *B. vulgatus* ATCC 8482 genomic DNA using *PfuTurbo* DNA polymerase (Stratagene) and I-­PIPE (Insert) primers (forward primer, 5′-ctgtacttccagggcGCGG­ATGATGACAAACCTATTCAAG-3′; reverse primer, 5′-aattaagtc­gcgttaATTGTCAATATCAATCACATTGAACTGC-3′; the target sequence is shown in upper case) that included sequences for the predicted 5′ and 3′ ends. The expression vector pSpeedET, which encodes an amino-terminal tobacco etch virus (TEV) protease-cleavable expression and purification tag (MGSDKIHHHHHH­ENLYFQ/G), was PCR-amplified with V-PIPE (Vector) primers (forward primer, 5′-taacgcgacttaattaactcgtttaaacggtctccagc-3′; reverse primer, 5′-gccc­tggaagtacaggttttcgtgatgatgatgatgatg-3′). V-PIPE and I-­PIPE PCR products were mixed to anneal the amplified DNA fragments together. *Escherichia coli* GeneHogs (Invitrogen) com­petent cells were transformed with the V-PIPE/I-PIPE mixture and dispensed onto selective LB–agar plates. The cloning junctions were confirmed by DNA sequencing. Using the PIPE method, the part of the gene encoding residues Met1–Trp19 (predicted signal sequence) was deleted. Expression was performed in selenomethionine-containing medium at 310 K. Selenomethionine was incorporated *via* inhibition of methionine biosynthesis (Van Duyne *et al.*, 1993 ▶), which does not require a methionine-auxotrophic strain. At the end of fermentation, lysozyme was added to the culture to a final concentration of 250 µg ml^−1^ and the cells were harvested and frozen. After one freeze–thaw cycle, the cells were homogenized in lysis buffer [50 m*M* HEPES pH 8.0, 50 m*M* NaCl, 10 m*M* imidazole, 1 m*M* tris(2-car­boxyethyl)phosphine–HCl (TCEP)] and the lysate was clarified by centrifugation at 32 500*g* for 30 min. The soluble fraction was passed over nickel-chelating resin (GE Healthcare) pre-equilibrated with lysis buffer, the resin was washed with wash buffer [50 m*M* HEPES pH 8.0, 300 m*M* NaCl, 40 m*M* imidazole, 10%(*v*/*v*) glycerol, 1 m*M* TCEP] and the protein was eluted with elution buffer [20 m*M* HEPES pH 8.0, 300 m*M* imidazole, 10%(*v*/*v*) glycerol, 1 m*M* TCEP]. The eluate was buffer-exchanged with TEV buffer (20 m*M* HEPES pH 8.0, 200 m*M* NaCl, 40 m*M* imidazole, 1 m*M* TCEP) using a PD-10 column (GE Healthcare) and incubated with 1 mg TEV protease per 15 mg of eluted protein. The protease-treated eluate was run over nickel-chelating resin (GE Healthcare) pre-equilibrated with HEPES crystallization buffer (20 m*M* HEPES pH 8.0, 200 m*M* NaCl, 40 m*M* imidazole, 1 m*M* TCEP) and the resin was washed with the same buffer. The flowthrough and wash fractions were combined and concentrated by centrifugal ultrafiltration (Millipore) to 9.7 mg ml^−1^ for crystallization trials. BVU2987 was crystallized using the nanodroplet vapor-diffusion method (Santarsiero *et al.*, 2002 ▶) with standard Joint Center for Structural Genomics (JCSG; http://www.jcsg.org) crystallization protocols (Lesley *et al.*, 2002 ▶). Sitting drops composed of 200 nl protein solution mixed with 200 nl crystallization solution were equilibrated against a 50 µl reservoir at 277 K for 37 d prior to harvesting. The crystallization reagent con­sisted of 35.0%(*v*/*v*) 2-­ethoxyethanol and 0.1 *M* cacodylate pH 6.5. No further cryoprotectant was added to the crystals. Initial screening for diffraction was carried out using the Stanford Automated Mounting system (SAM; Cohen *et al.*, 2002 ▶) at the Stanford Synchrotron Radiation Lightsource (SSRL). A rod-shaped crystal of approximate size 20 × 20 × 100 µm was harvested for data collection. The diffraction data were indexed in the orthorhombic space group *P*2_1_2_1_2_1_. To determine its oligomeric state in solution, BVU2987 was analyzed using a 1 × 30 cm Superdex 200 size-exclusion column (GE Healthcare) coupled with miniDAWN static light-scattering (SEC/SLS) and Optilab differential refractive-index detectors (Wyatt Technology). The mobile phase consisted of 20 m*M* Tris pH 8.0, 150 m*M* NaCl and 0.02%(*w*/*v*) sodium azide. The molecular weight was calculated using *ASTRA* v.5.1.5 software (Wyatt Technology).

### 2.2. Data collection, structure solution and refinement

Multi-wavelength anomalous diffraction (MAD) data were collected to 1.85 Å resolution on beamline 11-1 at SSRL at wavelengths corresponding to the high-energy remote (λ_1_), inflection point (λ_2_) and peak (λ_3_) of a selenium MAD experiment using the *Blu-Ice* data-collection environment (McPhillips *et al.*, 2002 ▶). A beam size of 0.15 × 0.15 mm was used during data collection. The λ_1_ and λ_2_ data sets were collected simultaneously interleaved in 30° wedges and were followed by λ_3_ (González, 2003*a* ▶,*b* ▶). The data set was collected at 100 K using a MarMosaic 325 CCD detector (Rayonix). The MAD data were integrated and reduced using *MOSFLM* (Leslie, 1992 ▶) and scaled with the program *SCALA* (Collaborative Computational Project, Number 4, 1994 ▶).

The heavy-atom sites were located with *SHELXD* (Sheldrick, 2008 ▶) and phasing was performed with *autoSHARP* (Vonrhein & Blanc, 2007 ▶). The heavy-atom substructure contained four anomalous scatterers per asymmetric unit, with an overall figure of merit (acentric/centric) of 0.39/0.33 and an anomalous phasing power for the three wavelengths of ∼0.5–0.8. *ARP*/*wARP* (Langer *et al.*, 2008 ▶) was used for automatic model building. Model completion and crystallo­graphic refinement were performed with the λ_1_ data set using *Coot* (Emsley & Cowtan, 2004 ▶) and *REFMAC*5 (Collaborative Computational Project, Number 4, 1994 ▶), respectively, with one TLS group per molecule (Winn *et al.*, 2003 ▶). Crystallographic data and refinement statistics are summarized in Table 1 ▶.

### 2.3. Validation and deposition

**Figure 1:**
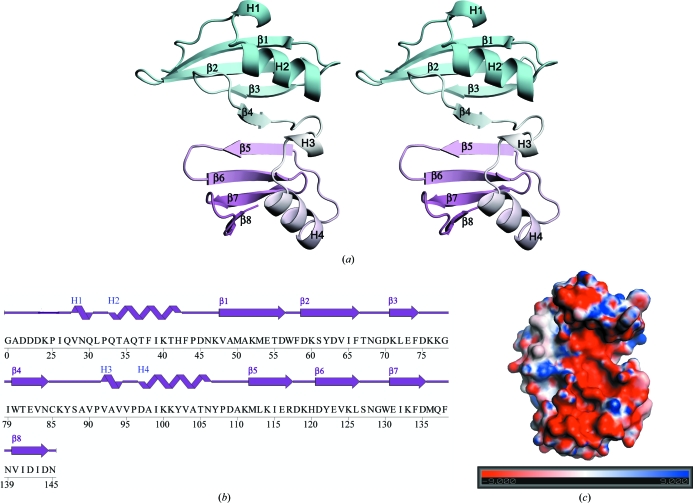
Crystal structure of BVU2987 from *B. vulgatus*. (*a*) Stereo ribbon diagram of the BVU2987 monomer with the N-terminal domain in cyan and the C-terminal tandem-repeat domain in pink. Helices H1–H4 (helices H1 and H3 are 3_10_-helices and helices H2 and H4 are α-helices) and β-strands β1–β8 are indicated. (*b*) Diagram showing the secondary-structural elements of BVU2987 superimposed on its primary sequence. The α-helices, 3_10_-helices and β-strands are indicated. The crystallized protein (including residues 20–145) was expressed with a tag that was removed during purification, leaving Gly0 followed by the target sequence (starting from residue 20). (*c*) The electrostatic surface potential reveals a prominent negatively charged region on the concave side of BVU2987 arising from the presence of numerous aspartic acid and glutamic acid residues (Asp21, Asp22, Glu54, Asp56, Asp59, Asp63, Glu73, Glu82, Glu116, Asp118, Glu123, Glu131, Asp142 and Asp144). The color scale is in units of ±*kT*/*e*.

The quality of the crystal structure was analyzed using the JCSG Quality Control server (http://smb.slac.stanford.edu/jcsg/QC). This server automatically processes the coordinates and data through a variety of validation tools including *AutoDepInputTool* (Yang *et al.*, 2004 ▶), *MolProbity* (Lovell *et al.*, 2003 ▶), *WHAT IF* v.5.0 (Vriend, 1990 ▶), *RESOLVE* (Terwilliger, 2003 ▶) and *MOLEMAN*2 (Kleywegt, 2000 ▶), as well as several in-house scripts, and summarizes the results. Protein quaternary-structure analysis was performed using the *PISA* server (Krissinel & Henrick, 2005 ▶). Fig. 1 ▶(*b*) was adapted from an analysis using *PDBsum* (Laskowski *et al.*, 2005 ▶) and all other figures were prepared with *PyMOL* (DeLano, 2008 ▶). Atomic coordinates and experimental structure factors for BVU2987 were deposited in the PDB under the accession code 3due. Fig. 1 ▶(*c*) was prepared using the *PDB*2*PQR* server (Dolinsky *et al.*, 2007 ▶) and the *APBS* module (Dolinsky *et al.*, 2007 ▶; Baker *et al.*, 2001 ▶) in *PyMOL*.

## 3. Results and discussion

### 3.1. Overall structure

The structure of BVU2987 was determined by MAD phasing to 1.85 Å resolution. The crystallized protein contained residues 20–145 of the full-length protein and an N-terminal glycine (that remained after the cleavage of the expression and purification tag). A predicted signal sequence (residues 1–19) was identified at the N-terminus of the full-length sequence and was omitted from the construct used for protein production. The final model contained one monomer, one cacodylate anion (from the crystallization condition) and 133 water molecules in the asymmetric unit. The Matthews coefficient (Matthews, 1968 ▶) is ∼2.3 Å^3^ Da^−1^, with an estimated solvent content of ∼47%. The Ramachandran plot produced by *MolProbity* (Davis *et al.*, 2004 ▶) showed that 96.8% and 100% of amino acids were in the favored and allowed regions, respectively. Crystal-packing analysis using *PISA* (Krissinel & Henrick, 2005 ▶), in addition to analytical size-exclusion chromatography coupled with static light scattering, indicated that the monomer was the favored oligomeric form in solution.

BVU2987 forms a crescent-shaped molecule comprised of an eight-stranded antiparallel β-sheet with four helices (two α-helices and two 3_10_-helices; Fig. 1 ▶ *a*). The β-sheet forms the inner concave side of the crescent and the helices form the outer edge of this ∼40 Å long and ∼30 Å wide molecule. The monomer is formed by a tandem repeat of a structural motif, possibly arising from a gene-duplication event, comprised of four antiparallel β-strands and a short helix–loop–long helix. Thus, residues 28–85 and 92–145 can be superimposed with an r.m.s.d. of 1.7 Å and a sequence identity of 22% over 54 aligned C^α^ atoms. The β-strands β1 and β2 in the first structural motif are slightly longer than the corresponding structural elements β5 and β6 in the tandem repeat, whereas β3 is slightly shorter than β7 (Fig. 1 ▶ *b*). Inspection of the electrostatic surface potential (Fig. 1 ▶ *c*) reveals that the concave surface has a prominent overall negative charge, mainly owing to the presence of numerous aspartic and glutamic acid residues.

Multiple orthologs of this protein family were targeted in parallel for structure determination; the crystal structures of two other proteins from this family were also determined and will be briefly described here. The structure of BT0923 (UniProt Q8A994; PDB code 3db7) from *B. thetaiotaomicron* VPI-5482 was determined at 1.40 Å resolution and that of BVU2443 (UniProt A6L337; PDB code 3elg) from *B. vulgatus* ATCC 8482 was determined at 1.64 Å resolution; these proteins have 73 and 42% sequence identity to BVU2987, respectively. These proteins are both very similar and superimpose on BVU2987 with r.m.s.d.s of 1.1 Å (over 122 aligned C^α^ residues) and 1.7 Å (over 119 aligned C^α^ residues), respectively.

### 3.2. Sequence and structural comparisons

Detailed sequence and structural analyses of the crystal structure of BVU2987 uncovered new relationships that unify the DUF2874 proteins into a superfamily of bacterial periplasmic proteins that includes PepSY, BLIP, SmpA_OmlA and DUF3192 proteins. Remote sequence similarities were first identified between DUF2874 and PepSY-domain proteins and were followed by sequence relationships between SmpA_OmlA, BLIP and DUF3192 that led to the identification of structural similarities between DUF2874, BLIP, SmpA_OmlA and PepSY proteins.

#### 3.2.1. Sequence relationship between DUF2874 and PepSY-domain proteins

After structure determination, sequence searches against protein-domain databases, such as Pfam (Finn *et al.*, 2008 ▶) and the Conserved Domain Database (CDD; Marchler-Bauer *et al.*, 2007 ▶), with BVU2987 did not find any significant hits. However, a *BLAST* (Altschul *et al.*, 1997 ▶) search revealed several related proteins (*E* value < 0.001). Regions that shared significant sequence similarity to either tandem repeat, as defined by the structure, were aligned using *MAFFT* (Katoh *et al.*, 2005 ▶) and the resulting multiple sequence alignment (representing a single domain) was used to construct a profile hidden Markov model (HMM) using the *HMMER* package (v.3.0, alpha release v.1.0). After multiple rounds of searching the UniProt sequence database (v.12.5) using the HMM, coupled with careful manual inspection of the resulting matches, we identified 271 sequences (*E* value < 0.01) which form a new protein family that has now been added to Pfam and appears in the new release (Pfam 24.0, October 2009) as DUF2874 (Pfam accession PF11396). These 271 DUF2874 domains are distributed in 153 distinct proteins from 40 species. In general, two copies of this domain are usually found in each protein, although single copies, and even up to four copies, also occur in some members of the family.

**Figure 2:**
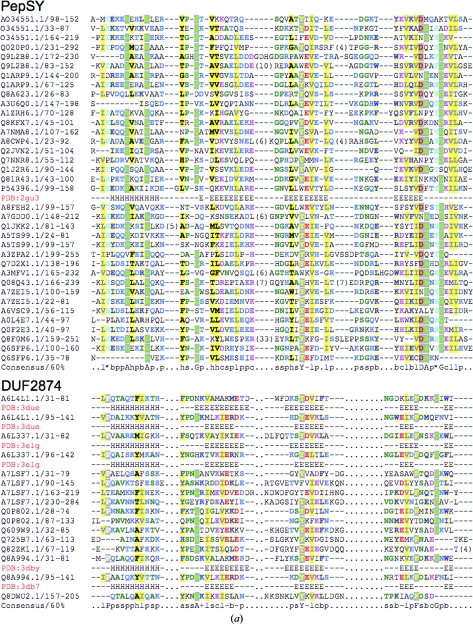

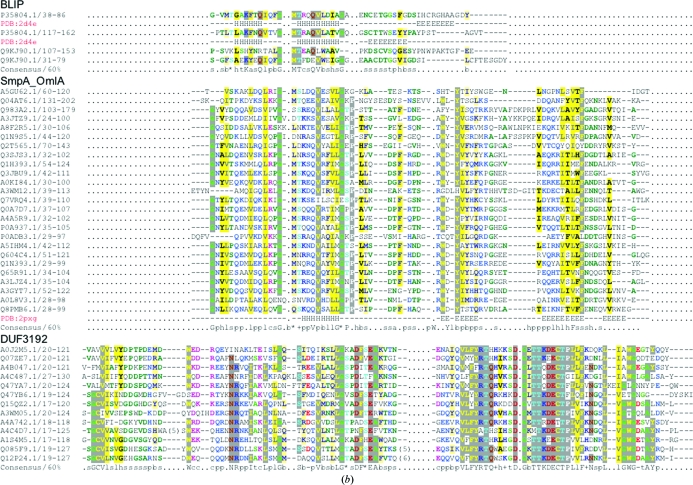
Alignments of representative multiple sequence alignments of the DUF2874 (Pfam accession PF11396), PepSY (PF03413), BLIP (PF07467), SmpA_OmlA (PF04355) and DUF3192 (PF11399) families. The alignments are colored according to the sequence conservation using *CHROMA* (Goodstadt & Ponting, 2001 ▶). The consensus sequence for the individual families calculated by *CHROMA* at a 60% threshold is shown under each alignment. Gaps inserted to maintain local sequence alignment are denoted with a ‘.’. Where appropriate, the PDB code and secondary structure is represented on a separate line under any sequence that has a known structure, with α-helical residues denoted ‘H’ and β-strand residues denoted ‘E’. The sequences, which are shown using the UniProt accession code, are representative both in terms of the sequence and species diversity. (*a*) Alignment of PepSY and DUF2487 families. (*b*) Alignment of the BLIP, SmpA_OmlA and DUF3192 families (*N.B.* the BLIP alignment is much shorter than DUF3192 as it is restricted to the conserved core of the domain).

Interestingly, the most significant marginal matches (*E*-value range 0.01–0.1, below the set threshold of 0.01) matched the HMM of the Pfam domain PepSY (Pfam accession PF03413). Inspection of these marginal hits suggested that PepSY-domain proteins were likely to be distant homologs of DUF2874. Profile–profile comparisons of all of the latest Pfam HMMs against each other (Madera, 2008 ▶) indicated significant similarity between the DUF2874 and PepSY families (*E* value of 5.7 × 10^−3^). The sequence relationship is demonstrated in the family pairwise sequence alignment in Fig. 2 ▶(*a*). In addition, the presence of a signal peptide motif (predicted using *PHOBIUS*; Kall *et al.*, 2004 ▶) at the N-terminus and the repetitive nature of the domain in some sequences are highly reminiscent of the domain architecture in the PepSY family (Yeats *et al.*, 2004 ▶). Unlike some members of the PepSY family where the PepSY domain co-occurs with other domains in the same protein (such as Peptidase_M4 and Peptidase_M36), no additional domains were found to co-occur in proteins containing DUF2874 domains. Further analysis was carried out to determine whether a single HMM could represent both DUF2874 and PepSY. However, a single model could not be built that was sufficiently sensitive to detect all of the domains that could be found using the two individual HMMs. This analysis demonstrates that PepSY and DUF2874 domains represent either a single divergent family or two related families of proteins that have arisen from a common evolutionary ancestor. Interestingly, the profile–profile comparisons also indicated that DsbC_N (Pfam accession PF10411), an N-terminal domain found in disulfide-bond isomerase (DsbC) proteins, may be related to DUF2874 (*E* value of 0.072). DsbC proteins not only function as disulfide-bond isomerases during oxidative protein folding in the bacterial periplasm, but have also been implicated as chaperones (Hiniker *et al.*, 2005 ▶). The structural representative of the DsbC_N family (PDB code 1t3b; Zhang *et al.*, 2004 ▶; aligns with BVU2987 with an r.m.s.d of 2.5 Å over 45 C^α^ atoms) is also found in the same SCOP fold as YpmB, a PepSY-family protein (PDB code 2gu3; J. Osipiuk, N. Maltseva, I. Dementieva, S. Moy & A. Joachimiak, unpublished work).

#### 3.2.2. Sequence relationship between SmpA_OmlA, BLIP and DUF3192 proteins

The recently determined first structural representative (PDB code 2pxg; Vanini *et al.*, 2008 ▶) of the SmpA_OmlA family of lipoproteins (Pfam accession PF04355) revealed structural similarity to BLIP (Pfam accession PF07467). As in BVU2987, each BLIP sequence contains a tandem repeat of a structural domain (four antiparallel β-strands and a short helix–loop–long helix), with the structure of OmlA being superimposable on both the N-terminal and C-­terminal copies of this domain (Vanini *et al.*, 2008 ▶). Given that the structurally equivalent positions between OmlA and BLIP corresponded to conserved residues among the BLIP sequences themselves, we took the Pfam BLIP HMM model from release 23.0 (which represented BLIP as a continuous sequence rather than a domain representing the tandem duplication) and modified it to represent the repeated domain. A single search using this modified BLIP HMM detected sequences from the SmpA_OmlA family, which highlighted the presence of a common evolutionary ancestor. This updated version of the BLIP family also appears in the new release of the Pfam database (Pfam 24.0, October 2009).

Profile–profile comparisons were again used to identify additional related families. These comparisons demonstrated that BLIP and SmpA_OmlA are significantly similar (*E* value of 2.8 × 10^−8^) and that both of these domains are also related to DUF3192 (Pfam accession PF11399), with *E* values of 5.4 × 10^−5^ for BLIP and 8.6 × 10^−5^ for SmpA_OmlA. Representative sequence alignments of each family over a similar region of the proteins (Figs. 2 ▶ *a* and 2 ▶ *b*) demonstrate the sequence conservation between PepSY and DUF2874 and between SmpA_OmlA, BLIP and DUF3192.

#### 3.2.3. Structural relationship between DUF2874, BLIP, SmpA_OmlA and PepSY proteins

**Figure 3:**
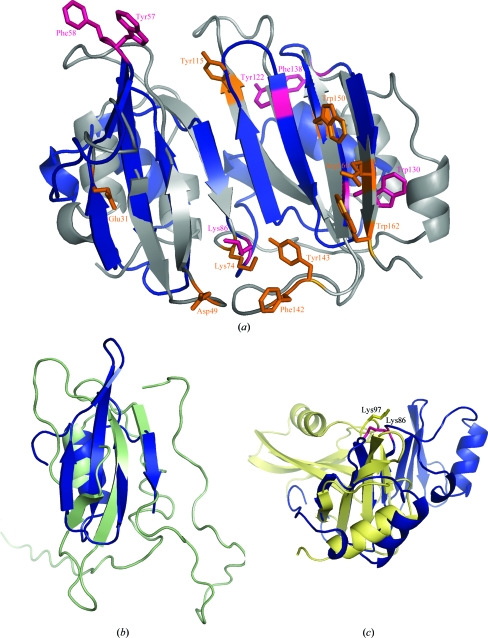
Structural comparisons of BVU2987 with related proteins. (*a*) Comparison of BVU2987 (blue) with BLIP (gray). The sequence conservation between the two proteins is <10% and among the functionally important residues (orange sticks) in BLIP only Lys74 is conserved in BVU2987 as Lys86 (magenta). Some of the important BLIP residues are aromatic residues that are present in loops that do not have counterparts in BVU2987. However, a few aromatic residues are present in other loops in BVU2987 (pink sticks) that could be functionally important. (*b*) Comparison of BVU2987 (N-terminal domain, blue) with OmlA protein (green; PDB code 2pxg). (*c*) Comparison of BVU2987 (blue) with YpmB (PepSY-domain protein; yellow; PDB code 2gu3). Lys86 of BVU2987 (magenta) is conserved as Lys97 in YpmB (yellow) and is structurally equivalent to Lys74 in BLIP, which is important in protein–protein interactions.

A systematic search for other proteins of similar structure to BVU2987 was conducted using several different methods including the *DALI* server (Holm *et al.*, 2008 ▶), the protein structure-comparison service *SSM* at the European Bioinformatics Institute (http://www.ebi.ac.uk/msd-srv/ssm; Krissinel & Henrick, 2005 ▶) and the flexible structure-alignment method implemented in *FATCAT* (Ye & Godzik, 2004 ▶). The most prominent hit was to BLIP (SCOP superfamily 55648 and SCOP fold 55647) from *Streptomyces clavuligerus* (UniProt BLIP_STRCL), for which structures are available in complex with *Klebsiella pneumoniae* SHV-1 β-­lactamase (PDB code 2g2u and related entries; Reynolds *et al.*, 2006 ▶), *E. coli* β-­lactamase TEM-1 (PDB code 1jtg and the related entries 1s0w and 1xxm; Strynadka *et al.*, 1996 ▶) and a putative BLIP from *Streptococcus mutans* (PDB code 3d4e; Joint Center for Structural Genomics, unpublished work). In the current Pfam PF07467/BLIP family, only three protein sequences are present from two species: BLIP_STRCL and P97062_STRCL from *Streptomyces clavuligerus* (with ∼31% sequence identity to each other) and Q9KJ90_STREX from *Streptomyces exfoliatus* (with ∼37% sequence identity to BLIP_STRCL). BLIP inhibits a wide variety of β-lactamases (such as TEM-1, which is the most widespread resistance enzyme to penicillin antibiotics). BLIP_STRCL is larger than BVU2987 by about 50 residues, although it also has an N-­terminal signal sequence and is a secreted protein. BVU2987 matches the different BLIP structures with *DALI Z* scores of 5.5–6.5 and with r.m.s.d.s of 2.7–3.4 Å over ∼75% of the residues (Fig. 3 ▶ *a*). The antiparallel β-sheet is conserved, although differences are found in the size of the connecting loops and in the positioning of the N-­terminal helices. The loop between the two tandem structural repeats is ∼10 residues long in BLIP and may contribute to its binding flexibility and its ability to inhibit a variety of class A β-­lactamases (Strynadka *et al.*, 1996 ▶). This loop is of similar length in BVU2987 and may confer similar flexibility.

Some of the important residues that have been implicated in the interactions of BLIP with SHV-1 β-lactamase (Reynolds *et al.*, 2006 ▶) are Glu31, Asp49, Lys74, Tyr115, Phe142, Tyr143, Trp150, Arg160 and Trp162 (numbering after removal of the N-terminal signal sequence). From the structural superposition (Fig. 3 ▶ *a*), only Lys74 in BLIP is conserved in BVU2987 as Lys86. Tyr115, Phe142 and Tyr143 in BLIP are located in long loops. Although these aromatic residues are not conserved in BVU2987, other aromatic residues (Trp57, Phe58, Tyr122, Trp130 and Phe138) are present in the corresponding shorter loops in BVU2987, which may be functionally important.

The concave surface of BVU2987 is negatively charged (Fig. 1 ▶ *b*) owing to the presence of numerous aspartate and glutamate residues. In contrast, the concave surface of BLIP has numerous uncharged polar residues (Ser35, Ser39, Tyr50, Tyr51, Tyr53, Thr55, Ser69, Ser71, Thr110, Ser113, Ser128, Ser130 and Ser146). Of these, Ser39 and Ser69 are conserved in BVU2987 as Ser61 and Thr81, respectively. This surface of BLIP also includes Phe36, His41, Trp112, His148, Trp150 and Trp162. It is interesting that the aromatic residues Tyr53, Trp112 and Trp150 in BLIP are structurally equivalent to the basic residues Lys71, Lys114 and Lys133 in BVU2987, respectively. It is possible that the long aliphatic tail of the lysine residues may mimic certain aspects of the hydrophobic tyrosine and tryptophan residues. Loop L_23_ between strands β2 and β3 (residues 46–51) in the first domain of BLIP is functionally important as Asp49 interacts with four conserved active-site residues in TEM-1 β-lactamase (Strynadka *et al.*, 1996 ▶), mimicking the interaction with the carboxylate group of its substrate penicillin G. The corresponding loop in BVU2987 is significantly shorter and is comprised of only two residues, 67–68. Interestingly, BLIP is similar to the TATA-box-binding protein in that it uses a tandem repeat of a structural motif of antiparallel β-­strands to create a concave saddle-shaped surface that can bind to a convex interacting partner (β-lactamase and DNA, respectively; Strynadka *et al.*, 1996 ▶). For BVU2987, the negatively charged concave surface is most likely to reflect binding to a positively charged partner.

It has recently been shown that members of the OmlA (outer membrane lipoprotein A) family are involved in the assembly of outer membrane proteins and in maintaining the structure of the cell envelope (Sklar *et al.*, 2007 ▶), although the actual mechanism is unknown. The structures of the BLIP-like domains of BVU2987 (residues 28–85) and the OmlA protein (PDB code 2pxg) superimpose with a *Z* score of 0.9 and an r.m.s.d. of 2.6 Å over 35 C^α^ atoms with 9% sequence identity (Holm & Park, 2000 ▶; Fig. 3 ▶ *b*). Although the *Z* score is below the standard significance cutoff of 2.0, OmlA nevertheless has a BLIP-like fold (Vanini *et al.*, 2008 ▶). Of the con­served N-terminal QGN motif and the four aromatic residues in the protein core that are seen in all OmlA proteins, only a single residue, Phe74 (equivalent to Phe76 in OmlA), is found in BVU2987.

The BLIP-like domains of BVU2987 and YpmB (a member of the PepSY family; PDB code 2gu3) superimpose with a *Z* score of 5.2 and an r.m.s.d. of 2.9 Å over 58 C^α^ atoms with 9% sequence identity, but the relative orientation of the tandem structural repeats in the two proteins are different (Fig. 3 ▶ *c*). Interestingly, Lys86 of BVU2987, which is the counterpart of the functionally important Lys74 in BLIP (mutation of this residue causes disruption of the BLIP–β-lactamase interface), is present as Lys97 in YpmB. Although the PepSY and DUF2874 domains appear to be more closely related by sequence, structural analysis indicates a greater similarity of DUF2874 to BLIP. This may account for the discrepancy in the SCOP classification where YpmB has been classified under a different SCOP fold, 54402.

### 3.3. Potential function based on similarity to related families

**Figure 4:**
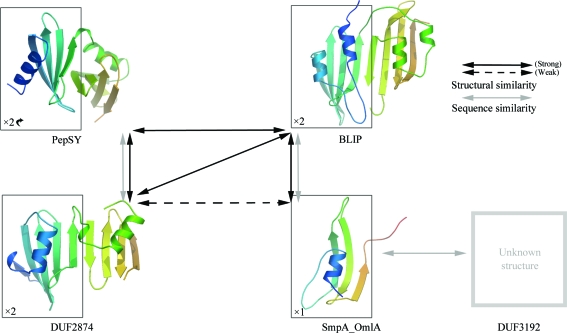
Schematic of the structural and sequence relationships between families belonging to the BLIP-like superfamily. PepSY, DUF2487, BLIP and SmpA_OmlA structures were rendered using *OpenAstexViewer* (Hartshorn, 2002 ▶). The structure is colored from blue at the N-terminus to orange at the C-terminus. For each family, a box indicates the portion of the representative structure that corresponds to a single copy of the domain. The tandem domain arrangement is obvious for DUF2847 and BLIP, but less so for PepSY owing to the different rotation of the second domain with respect to the first copy of the domain. SmpA_OmlA only has a single copy of the domain. No three-dimensional structure is known for DUF3192. Relationships between the families that could be identified using sequence-based methods only are shown as gray arrows, whereas strong structural similarity is indicated by solid black arrows and weak structural similar by dashed black arrows.

We have identified five bacterial periplasmic protein domain families (DUF2874, PepSY, BLIP, SmpA_OmlA and DUF3192) that are related by sequence and/or structural similarity (Fig. 4 ▶). BLIP binds to numerous class A β-lactamases and prevents them from hydrolyzing β-lactam antibiotics. Gene-knockout studies of BLIP in *Streptomyces exfoliatus* SMF19 have indicated that BLIP may have a broader role, particularly in regulating cell morphology (Kang *et al.*, 2000 ▶), which is thought to be mediated by its binding to penicillin-binding proteins involved in cell-wall synthesis. Apart from BLIP, the precise functions of these other families remain to be elucidated. Nevertheless, a number of recurring themes appear to be emerging.

The PepSY domain, when found in combination with other Pfam domains, is typically associated with M4 or M36 peptidases. These peptidases all function in the periplasmic space and it has been postulated that the PepSY domain functions as an inhibitor of the peptidase. The same PepSY domain is also found in YpmB, which is co-expressed with SleB (Boland *et al.*, 2000 ▶). In this case, SleB, a lytic enzyme, is inhibited by YpmB; given the lack of any sequence similarity between the peptidase and this lytic peptide, it has been suggested that PepSY may also function as a broad-spectrum inhibitor (Yeats *et al.*, 2004 ▶).

PepSY and DUF2874 domains are found in most protein sequences where no other associated domains are present. The precise function of OmlA remains unclear, but it is thought to be involved in maintaining the integrity of the cell envelope (Ochsner *et al.*, 1999 ▶). A knockout study in *Xanthomonas campestris* pv. *phaseoli* indicated that even though OmlA is divergently transcribed from the gene encoding the ferric uptake regulator Fur, the absence of Fur does not alter OmlA expression. In the same study, an OmlA mutant showed increased susceptibility to novobiocin and coumermycin, which are antibiotics with gyrase inhibitory activity. How OmlA protects the cell against these antibiotics or maintains the cell envelope is not known, but given the similarity to BLIP it is interesting to speculate that a similar inhibitory/regulatory binding mechanism may be employed in these two cases.

The BVU2987 structure is the first structural representative of a novel protein family, which has now been added to the Pfam database as DUF2874. The sequence and structural analyses presented show that this family is a member of a superfamily containing four other related bacterial periplasmic protein families: PepSY, BLIP, SmpA_OmlA and DUF3192. The protein structures from these families all adopt a BLIP-like fold. Although the precise functions of PepSY, DUF2874, SmpA_OmlA and DUF3192 remain to be elucidated, it seems that they function as inhibitors by binding a partner domain located either on the same protein or on a separate protein. The structure of BVU2987 reveals an internal duplication of a domain that occurs between one and four times in different sequences. BLIPs are important for the design of peptide-based β-lactamase inhibitors and for studying protein–protein interactions. Thus, the similarity between these families opens up the possibility of biochemical studies and therapeutic potential. Members of the DUF2874 family define a new type of BLIP-like protein produced by the human gut microbiome. The structures of DUF2874 presented here can be used to investigate whether these proteins do indeed inhibit β-lactamases of the human gut (Chanal *et al.*, 1996 ▶). If so, these different BLIP-like proteins could be utilized in the design of novel peptide-like β-­lactamase inhibitors.

Additional information about BVU2987 is available from TOPSAN (Krishna, 2010 ▶) at http://www.topsan.org/explore?PDBid=3due.

## Supplementary Material

PDB reference: BVU2987, 3due

## Figures and Tables

**Table 1:** Crystallographic data and refinement statistics for BVU2987 (PDB code 3due) Values in parentheses are for the highest resolution shell.

	λ_1_ MAD-Se	λ_2_ MAD-Se	λ_3_ MAD-Se
Data collection			
Space group	*P*2_1_2_1_2_1_		
Unit-cell parameters (Å)	*a* = 31.60, *b* = 50.87, *c* = 79.51		
Wavelength (Å)	0.9184	0.9793	0.9788
Resolution range (Å)	29.4–1.85 (1.90–1.85)	29.4–1.85 (1.90–1.85)	29.4–1.85 (1.90–1.85)
No. of observations	40890	40928	41013
No. of unique reflections	11519	11544	11557
Completeness (%)	100.0 (100.0)	100.0 (100.0)	100.0 (100.0)
Mean *I*/σ(*I*)	8.8 (2.0)	9.2 (2.0)	8.7 (1.6)
*R*_merge_ on *I*† (%)	10.9 (58.8)	10.4 (56.9)	11.2 (69.6)
*R*_meas_ on *I*‡ (%)	12.9 (69.2)	12.2 (67.0)	13.2 (82.0)
Model and refinement statistics			
Resolution range (Å)	29.4–1.85		
No. of reflections (total)	11480§		
No. of reflections (test)	549		
Completeness (%)	99.96		
Data set used in refinement	λ_1_		
Cutoff criteria	|*F*| > 0		
*R*_cryst_¶	0.192		
*R*_free_¶	0.233		
Stereochemical parameters			
Restraints (r.m.s.d. observed)			
Bond angles (°)	1.65		
Bond lengths (Å)	0.015		
Average isotropic *B* value†† (Å^2^) (all atoms/protein residues only)	22.4/21.2		
ESU‡‡ based on *R*_free_ (Å)	0.14		
Protein residues/atoms	126/1037		
Waters/cacodylate	133/1		

† *R*_merge_ = 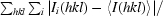 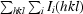.

‡ *R*_meas_ = 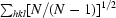 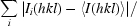 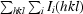 (Diederichs & Karplus, 1997 ▶).

§ Typically, the number of unique reflections used in refinement is slightly less than the total number that were integrated and scaled. Reflections are excluded owing to negative intensities and rounding errors in the resolution limits and unit-cell parameters.

¶ *R*_cryst_ = 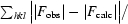 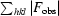, where *F*_calc_ and *F*_obs_ are the calculated and observed structure-factor amplitudes, respectively. *R*_free_ is the same as *R*_cryst_ but for 4.9% of the total reflections chosen at random and omitted from refinement.

†† This value represents the total *B* that includes TLS and residual *B* components.

‡‡ Estimated overall coordinate error (Collaborative Computational Project, Number 4, 1994 ▶; Cruickshank, 1999 ▶).
